# Deciphering the interplay between fruit-associated metabolites and bacterial communities across four distinct mango cultivars

**DOI:** 10.3389/fpls.2026.1754579

**Published:** 2026-02-06

**Authors:** Chuanfang Zhang, Rong Wan, Siwei Nong, Wei Huang, Salim S. Al-Rejaie, Fengzhen Wang, Zhengzhou Yang, Zhengjie Zhu, Mohamed Mohany

**Affiliations:** 1 Guangxi Key Laboratory of Biology for Mango, College of Agriculture and Food Engineering, Baise University, Baise, China; 2 College of Subtropical Characteristics, Agricultural Industry, Baise, China; 3 Sichuan Vocational and Technical College, Suining, Sichuan, China; 4 Department of Pharmacology and Toxicology, College of Pharmacy, King Saud University, Riyadh, Saudi Arabia

**Keywords:** bacterial microbiota, fruit metabolome, fruit quality, fruit-metabolite-microbiome interactions, mango cultivars, postharvest biology

## Abstract

Mango (*Mangifera indica* L.) fruit characteristics and health are strongly determined by their biochemical profiles and fruit-associated microbiome composition. However, the cultivar-specific interplay between the mango fruit metabolome and microbiome remains elusive. Here, we tracked differential changes in fruit metabolites and bacterial community composition in four economically important mango cultivars in China: Qingmang (QM), Yumang (YM), Tainong (TN), and Aomang (AM). Using untargeted metabolomics with liquid chromatography-mass spectrometry and high-throughput amplicon sequencing of bacterial 16S rRNA, we identified distinct metabolic profiles and the enrichment of a specific subset of microbiota unique to each cultivar. Different metabolites associated with nitrogen and carbon metabolism, biosynthesis of amino acids, secondary metabolites, and flavonoids were differentially abundant in the four mango cultivars. These classes of metabolites have been previously linked to fruit development, color, antioxidant capacity, and stress resistance. Importantly, significant positive correlations were found between specific bacterial taxa, such as *Alcanivorax*, *Alistipes*, *Curtobacterium*, *Rikenella*, *Thiopseudomonas*, *Rikenella*, and *Vogesella* and the accumulation of the metabolites ornithine, L-arginine, tricetin, casoxin D, mhppa sulfate, sorbitan palmitate, meconic acid and rengyoside B. These results indicate the critical role of mango cultivars in shaping the fruit-specific microbiomes and metabolites. Our findings provide a foundational understanding of mango fruit holobionts and offer novel insights into metabolic and microbial networks for developing strategies to enhance fruit quality and postharvest management.

## 1 Introduction

Mango (*Mangifera indica* L.) is an important fruit tree crop with a vast genetic diversity, rich taste, and nutritional benefits. Each variety of mango exhibits distinct characteristics, including fruit size, color, aroma, and unique flavor, which represent their individual identities and appeals (Nassur et al., 2015). Importantly, the variation in mango fruit size from small to medium to large, as well as fruit skin color, such as yellow, orange, red, and combinations thereof, are potent factors influencing consumer preference and the market value of mango fruits (Dos Santos Moreira et al., 2024). Mango fruit characteristics are hypothesized to be strongly linked to the metabolic composition (Maldonado-Celis et al., 2019). Previous studies have demonstrated that mango fruit content, such as sugars, amino acids, organic acids, and volatile organic compounds, differs between mango cultivars and fluctuates with fruit development stage (Peng et al., 2022). Therefore, the use of metabolomic approaches, such as liquid chromatography-mass spectrometry (LC-MS), may allow us to understand changes in the composition of metabolites across different regional mango cultivars, which may help enhance flavor, nutritional quality, and postharvest stability.

Although the metabolome underpins the biochemical profile of fruit, microbial communities have been associated with fruit development, flavor evolution, and pathogen resistance (Droby and Wisniewski, 2018; Escobar Rodríguez et al., 2021; Wu et al., 2019). Numerous studies have indicated that fruit microbial diversity and composition are influenced by host genotype and fruit developmental stages (Liu et al., 2018; Zhimo et al., 2022). Interestingly, fruit microbial communities are involved in cross-talk with host metabolism to alter biochemical pathways that affect the ripening process (Kifle et al., 2024). These specific microbial assemblages within the fruit microbiome degrade host metabolites or synthesize novel metabolites, thereby affecting fruit flavor, causing disease suppression, and extending the shelf life (Kifle et al., 2024; Luo et al., 2024). Therefore, a detailed understanding of various mango cultivars harboring distinct bacterial microbiota is crucial for sustainable microbiome-based interventions to enhance fruit quality and health.

Recent advances in integrative approaches, combining metabolomics and microbiome profiling, offer an innovative approach to clarify the functional interplay between the host microbiota and metabolic phenotypes. The activity of plant microbiota can alter the composition of host secondary metabolites, including fruit flavor-associated metabolites, such as carotenoids and flavonoids (Robards and Antolovich, 1997; Kochevenko et al., 2012). For example, a specific subset of microbiota, such as methylotrophic bacteria and rhizobacteria, has been linked to the flavor of strawberry and rice (Verginer et al., 2010; Deshmukh et al., 2016). Moreover, a recent study found a dynamic link between fruit-associated bacterial microbiota and changes in fruit aroma and raspberry (Sangiorgio et al., 2021). Hundreds of aromatic volatiles are produced by different mango cultivars (Lalel et al., 2003a, 2003b), some of which are mainly emitted after glycoside hydrolysis during fruit storage (Sakho et al., 1997; Ubeda et al., 2012). In addition, mango fruit microbiota has been extensively explored in association with disease development. However, there is a lack of information on the relationship between mango metabolites and the fruit microbiota in different mango cultivars.

In this study, we investigated four different mango cultivars with varying skin colors for fruit metabolite signatures and associated microbiomes to characterize cultivar-specific profiles. We hypothesized that the relationship between the fruit microbiome and metabolites is specific to each mango cultivar, and that their dynamic interplay across cultivars contributes to differences in nutritional content and fruit quality. By integrating metabolomics with the composition of fruit microbiota, we aimed to uncover the relationship between host microbes and metabolite dynamics. The objectives of this study were to (1) determine the core and differential microbial taxa associated with the fruits of each mango cultivar; (2) quantify key and differential metabolites linked to flavor, aroma, and nutritional quality in each mango cultivar using untargeted metabolomics; and (3) assess significant correlations between specific microbial taxa and metabolite abundance across cultivars. The identification of specific microbial taxa and metabolites that reflect differences in fruit morphology and metabolism across cultivars would enhance our understanding of mango agroecosystems and support their sustainable production.

## 2 Materials and methods

### 2.1 Sample collection

Mature mango fruit samples were collected from Youjiang district of Baise City (23° 54’ 19.2996” N, 106° 36’ 53.5752” E), China between June and August 2024. The fruit belongs to the mango cultivars Tainong (TN), Aomang (AM), Qingmang (QM), and Yumang (YM). These cultivars were grown in distinct orchards within a 2 km radius of the Youjiang district, ensuring that the selected orchards were representative of the typical growing conditions within the region. In total, four independent trees were sampled; five fruits were collected from each tree, and the representative replicate from each tree was a composite of five fruits. The collected mango samples were transferred to plastic bags and immediately transported to the laboratory in a cool box. The samples collected from each cultivar provided representative coverage of the primary mango cultivar and sufficient fruit tissue for downstream analysis of metabolomics and microbiome sequencing.

### 2.2 Sample processing

The collected mango fruit samples were dissected for combined peel-pulp fractions using a sterilized scalpel, hereafter referred to as mango peel-pulp. Each replicate consisted of five composite fruit samples from each of the four trees for each cultivar, which were weighed equally before and ground using a ZT-1000A high-speed multifunctional grinder (Yongkang Zhanfan Industry and Trade Co., Ltd.) and further milled using a DLF-50S classified ultra-fine water-cooled grinder (Wenzhou Dingli Medical Instruments Co., Ltd.). The resulting powder was passed through a 32-mesh sieve and stored in vacuum-sealed drying bags at room temperature under anaerobic conditions for DNA extraction and metabolomic analysis.

### 2.3 Nutritional analysis of mango fruit

The moisture content of the mango peel-pulp was determined using a drying method, following the national standards of the People’s Republic of China GB/T 6435-201 (National Technical Committee of Feed Industry Standardization, 2014). Crude protein content was measured using the Kjeldahl nitrogen method according to the China National Standard GB/T 6432-2018 (National Technical Committee of Feed Industry Standardization, 2018). Crude fat content was analyzed by Soxhlet extraction using a SOX606 fat analyzer (Hanon Instruments Co. Ltd., Jinan, China) according to the China National Standard GB 5009.6-2016 (National Technical Committee of Feed Industry Standardization, 2016). The crude ash content was determined according to the China National Standard GB/T 6438-2007 (National Technical Committee of Feed Industry Standardization, 2007). Neutral detergent fiber content was determined using the crucible method GB/T 20806-2022 (National Technical Committee of Feed Industry Standardization, 2022a). Acid detergent fiber and lignin content were examined according to the China National Standard NY/T 1459-2022 (National Technical Committee of Feed Industry Standardization, 2022b). Soluble sugars were quantified using the 3,5-dinitrosalicylic acid colorimetric method according to the China National Standard NY/T 2742-2015 (National Technical Committee of Feed Industry Standardization, 2015).

### 2.4 Metabolomics analysis of mango fruits

For untargeted LC-MS analysis to profile metabolites from mango fruit, the mango peel-pulp samples were accurately weighed into 2 mL centrifuge tubes, and then 1 mL of tissue extraction solvent (75% methanol:chloroform 9:1, 25% water) was added along with steel beads for homogenization. The samples were homogenized by grinding at 50 Hz for 60 s in a tissue grinder. After tissue homogenization, the sample tubes were centrifuged at 12,000 rpm for 10 min and the resultant supernatant was transferred to a new tube. Chromatographic separation was performed on a Vanquish UHPLC system using an ACQUITY UPLC^®^ HSS T3 column (2.1 × 100 mm, 1.8 µm) at 40°C, with a flow rate of 0.3 mL/min and 2 µL injection volume. The mobile phase for the positive-ion mode consisted of 0.1% formic acid in acetonitrile and 0.1% formic acid in water. For the negative ion mode, acetonitrile and 5 mM ammonium formate were used with appropriate gradient elution programs. Mass spectrometry was performed on a Thermo Orbitrap Exploris 120 equipped with an ESI source operating in both positive and negative ion modes, with spray voltages of +3.50 kV and -2.50 kV, respectively, sheath gas at 40 arb, auxiliary gas at 10 arb, and capillary temperature at 325°C. The MS1 resolution was set to 60,000, scanning m/z 100–1000, with data-dependent MS/MS fragmentation at 30% normalized collision energy, and MS2 resolution of 15,000, employing dynamic exclusion to improve data quality.

The obtained raw data files were converted to the mzXML format using ProteoWizard, and peak detection, filtering, retention time correction, and alignment were performed using XCMS with parameters optimized for accurate feature extraction. Batch effects were corrected using support vector regression based on QC samples and features with QC relative standard deviations above 30% were excluded. Multivariate statistical analyses, including Principal Component Analysis (PCA), were conducted using the ropls package in R to distinguish sample groups and identify differential metabolites with model validation using permutation tests. Significant metabolites were selected based on VIP scores of >1 and p <0.05, as shown in the volcano plot. Metabolites were identified by matching accurate mass and MS/MS fragmentation patterns against the Kyoto Encyclopedia of Genes and Genomes (KEGG) and the Human Metabolome Database (HMDB). Pathway enrichment and topology analyses were performed using MetaboAnalyst, and the results were visualized using KEGG Mapper to interpret the biological relevance of differential metabolites.

### 2.5 DNA extraction and high-throughput amplicon sequencing

Bacterial 16S rRNA gene amplicon sequencing was performed using the Illumina HiSeq platform. DNA from peel-pulp samples of each mango fruit was extracted using the DNeasy PowerSoil Kit (QIAGEN, Hilden, Germany), and the concentration was measured using a NanoDrop2000 spectrophotometer (Thermo Fisher Scientific, Wilmington, DE, USA). The 16S rRNA gene amplicons were generated using primers V3-V4 region 335F (5’-CADACTCCTACGGGAGGC-3’) and 769R (5’-ATCCTGTTTGMTMCCCVCRC-3’) (Dorn-In et al., 2015). The USEARCH tool was used to process the generated paired-end Illumina reads, followed by joining the paired-end reads, relabeling the sequencing names, trimming the barcodes and primers, and filtering low- and high-quality reads. High-quality reads obtained were assigned to operational taxonomic units (OTUs) with 97% sequence similarity. The OTUs assigned to plant organelles were discarded. Taxonomic classification of the OTUs was performed against the SILVA database (Quast et al., 2013) using the RDP Classifier algorithm. All raw sequencing data from this project are available in the NCBI Sequence Read Archive (SRA) database under BioProject PRJNA1367298.

### 2.6 Bioinformatics analysis

Mango fruit bacterial community analysis was performed using R software (v4.5.1). Any sequences annotated as plant mitochondria or chloroplasts were removed prior to downstream analysis. The OTU table was rarefied to 90,915 sequences per sample based on the lowest number of sequences contained in a sample (min= 90,915; max=287,564). Alpha diversity including Shannon diversity and Simpson diversity index analysis of bacterial communities were performed using R package ‘vegan’. The treatment mean values of different mango organs were compared using analysis of variance (ANOVA) and least significant difference (LSD) test. Beta diversity analysis based on the unweighted Unifrac distance for the bacterial community was performed using the ‘UniFrac’ function in R-Package ‘phyloseq’. Principal coordinate analysis (PCoA) plots were constructed based on unweighted UniFrac distance. Permutational multivariate ANOVA was performed by ‘adonis’ function in R package ‘vegan’ to determine the significant differences in bacterial community composition across four mango cultivars. The unique and shared bacterial OTUs across the four cultivars were examined in a Venn diagram using the R package ‘VennDiagram’. The average RAs of bacterial phyla and families were calculated using OTUs and bar graphs were plotted by the R package ‘ggplot2’. Heatmaps were constructed to visualize the relative abundance of bacterial genera using the function heatmap.2 in the gplots. ANOVA and LSD tests were used to determine significant differences between the groups. The correlation between the bacterial microbiota and specific metabolites was determined using the R package ‘ggcorrplot’.

## 3 Results

### 3.1 Nutrient contents in the peel-pulp of different mango cultivars

**Figure 1:**
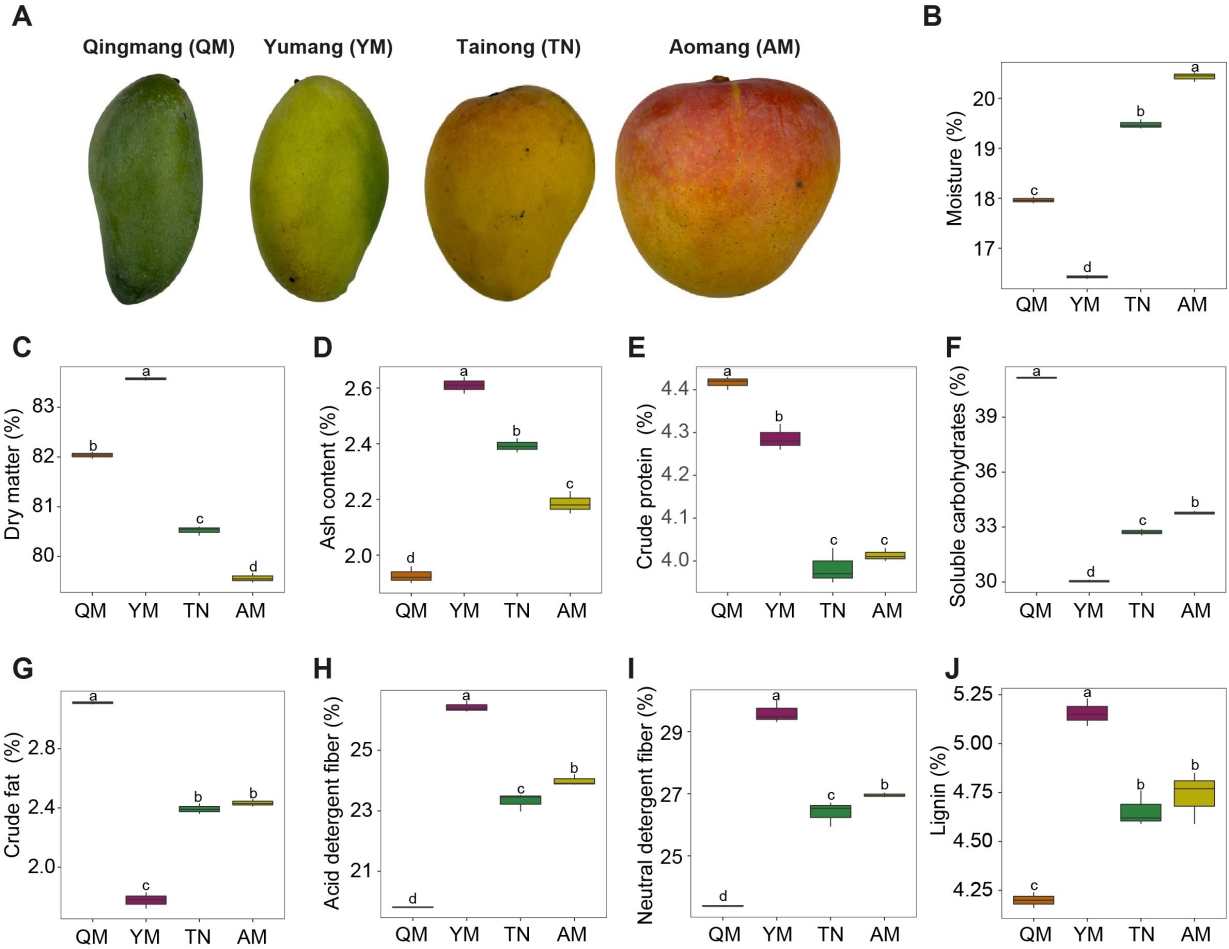
Composition of the different nutrients in peel-pulp of mango. **(A)** Representative images of mango fruits from four distinct cultivars. The analysis of mangoes moisture content **(B)**, dry matter **(C)**, ash **(D)**, crude protein **(E)**, soluble carbohydrates **(F)**, crude fat **(G)** acid detergent fiber **(H)**, natural detergent fiber **(I)**, and lignin content **(J)**. Whiskers in the boxes with different colors indicate the range of minimum and maximum value across four cultivars. Different letters above each box indicate statistically significant differences according to the LSD test (p < 0.05). QM, Qingmang; YM, Yumang; TN, Tainong; and AM, Aomang.

The nutritional composition of mango peel-pulp from the four cultivars was analyzed using standardized methods (**Figure 1A**). The moisture content was highest in Aomang (AM), followed by Tainong (TN), Qingmang (QM), and Yumang (YM) (**Figure 1B**). Dry matter and ash contents were highest in YM (**Figures 1C, D**). Crude protein, fat, and soluble carbohydrate contents were high in the QM cultivar (**Figures 1E–G**). In contrast, the YM cultivar had significantly lower soluble carbohydrate and fat content than the other cultivars. Acid detergent fiber, neutral detergent fiber, and lignin contents were highest in YM, followed by AM, TN, and QM mangoes (**Figures 1H–J**).

### 3.2 Metabolites composition in different mango cultivars

**Figure 2:**
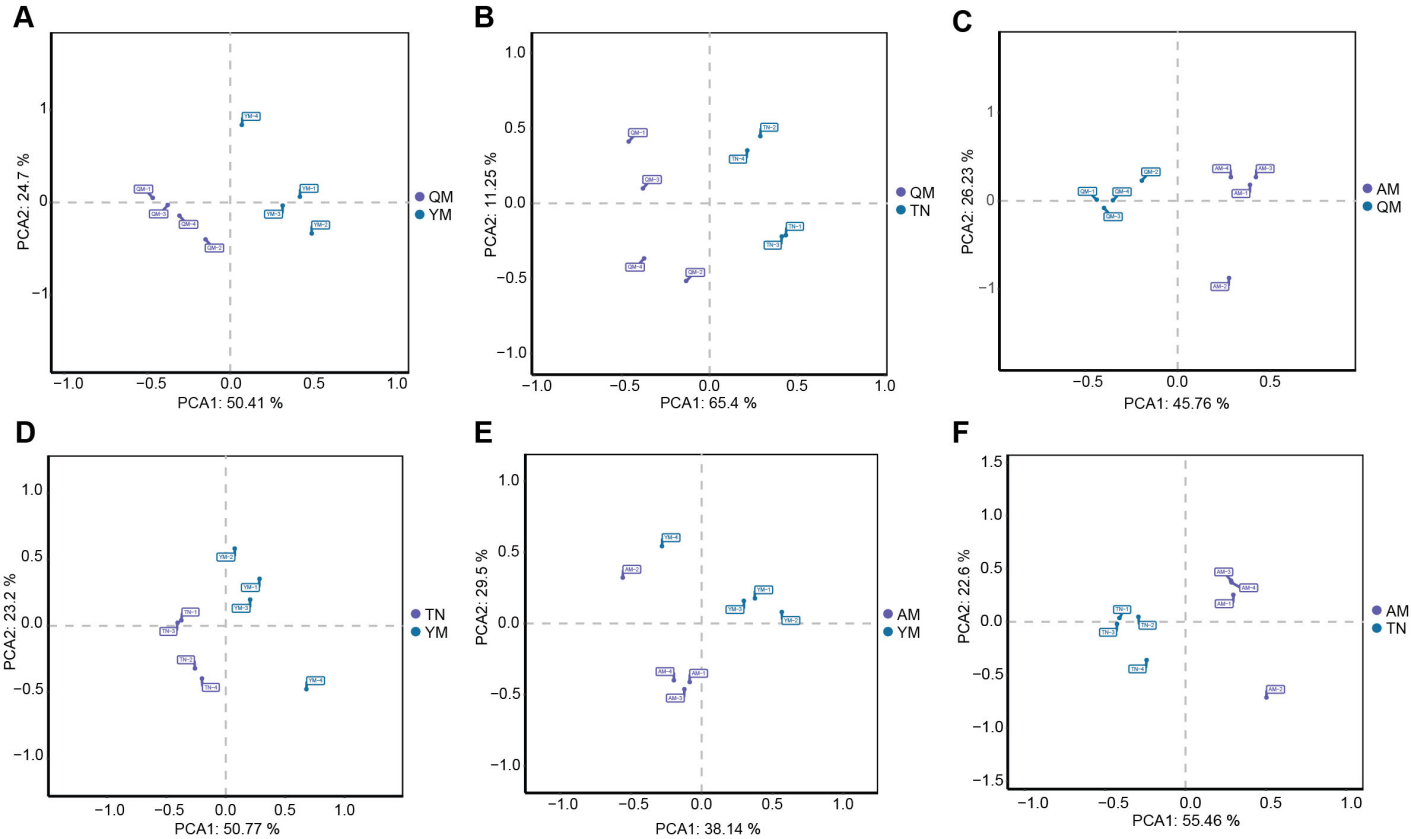
Principal component analysis (PCA) of mango fruit metabolites between cultivars. Pair-wise comparative analysis of mango metabolites in QM and YM **(A)**, QM and TN **(B)**, QM and AM **(C)**, YM and TN **(D)**, YM and AM **(E)**, and TN and AM **(F)**. QM, Qingmang; YM, Yumang; TN, Tainong; AM, Aomang.

A total of 2393 metabolites were detected in four distinct mango cultivars, TN, AM, QM, and YM, based on untargeted LC-MS analysis. All four cultivars showed signs of significant variation in metabolite composition within the principal component analysis (PCA) plots based on the overall clustering of mango samples (**Supplementary Figure S1**). We then performed pair-wise comparisons among the four cultivars to understand the variation in their metabolite composition. Mango fruits from all cultivars exhibited distinct metabolic profiles, as can be seen by sample clustering in the PCA plots (**Figures 2A–F**).

The QM and YM cultivars were clearly separated along PC1 (50.41%) and PC2 (24.7%), suggesting distinct metabolic profiles (**Figure 2A**). A clear separation between QM and TN was observed, with PC1 (65.4%) and PC2 (11.25%) explaining most of the variance (**Figure 2B**). AM and QM also showed distinct separation along PC1 (45.76%) and PC2 (23.53%), indicating differences in metabolic composition between cultivars (**Figure 2C**). The cultivars TN and AM, in comparison to YM, showed moderate but significant separation, indicating partial similarity in their metabolic composition (**Figures 2D, E**). Finally, the AM and TN cultivars also showed significant differences, with PC1 (55.46%) and PC2 (22.6%) explaining a significant portion of the variation. Overall, these results indicate a significant degree of metabolic distinctiveness between the different mango cultivars.

**Figure 3:**
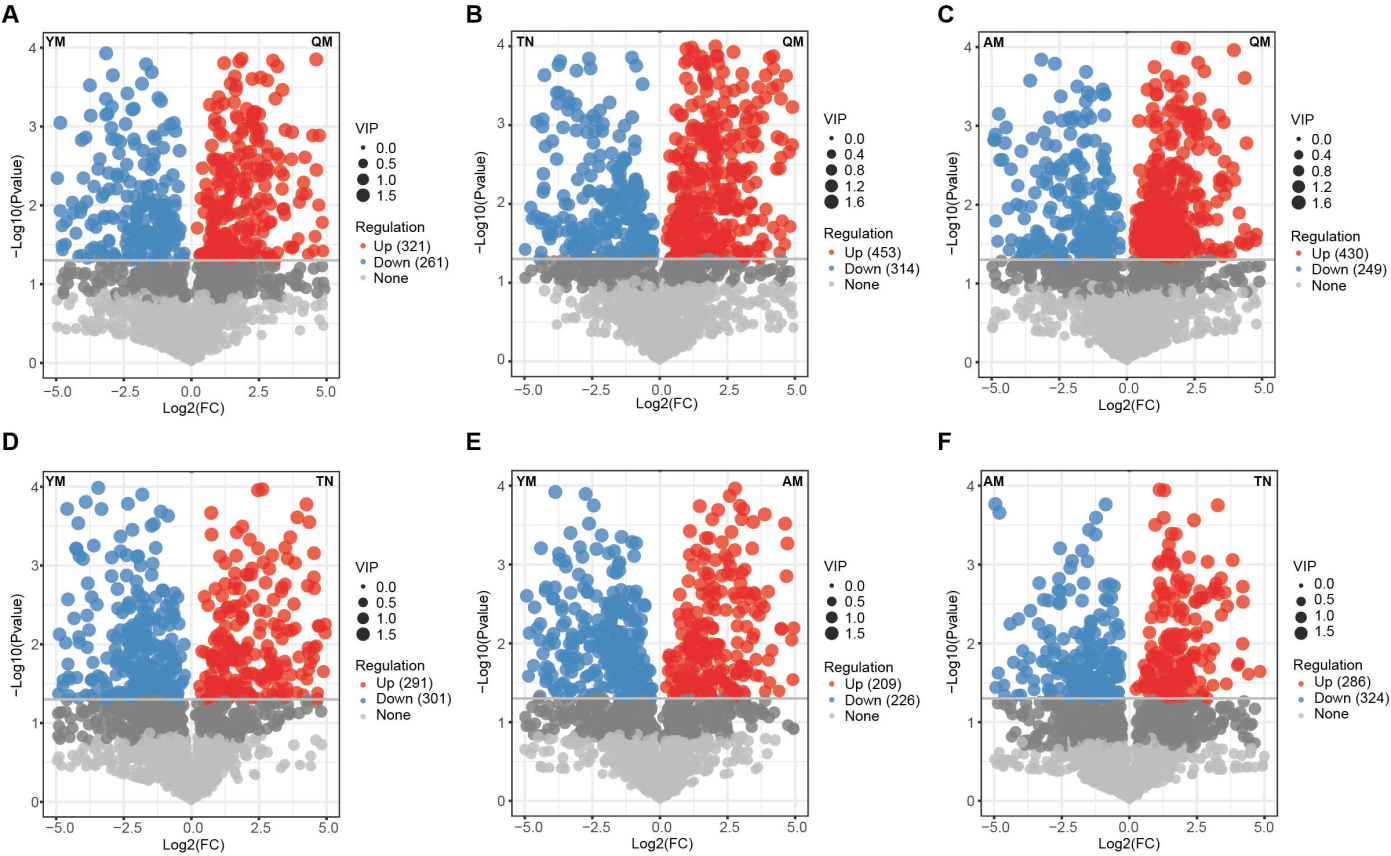
Pair-wise differentially abundant metabolites analysis across four mango cultivars. Volcano plots showing the differentially abundant metabolites between QM and YM **(A)**, QM and TN **(B)**, QM and AM **(C)**, YM and TN **(D)**, YM and AM **(E)**, and TN and AM **(F)**. QM, Qingmang; YM, Yumang; TN, Tainong; AM, Aomang.

Comparative analysis of metabolite composition between Qingmang and the other cultivars revealed significantly distinct metabolite enrichment, with Qingmang enriched in 321, 453, and 430 metabolites compared to Yumang, Tainong, and Aomang, respectively, while these cultivars showed enrichment of 261, 314, and 249 metabolites relative to Qingmang (**Figures 3A–C**). Similarly, the Yumang cultivar was enriched in 301 and 226 metabolites compared to Tainong and Aomang, respectively, whereas these cultivars showed enrichment in 291 and 209 metabolites relative to Yumang (**Figures 3D, E**). The Tainong cultivar was enriched in 286 metabolites compared with Aomang, whereas this cultivar showed an enrichment of 324 metabolites relative to Tainong (**Figure 3F**).

**Figure 4:**
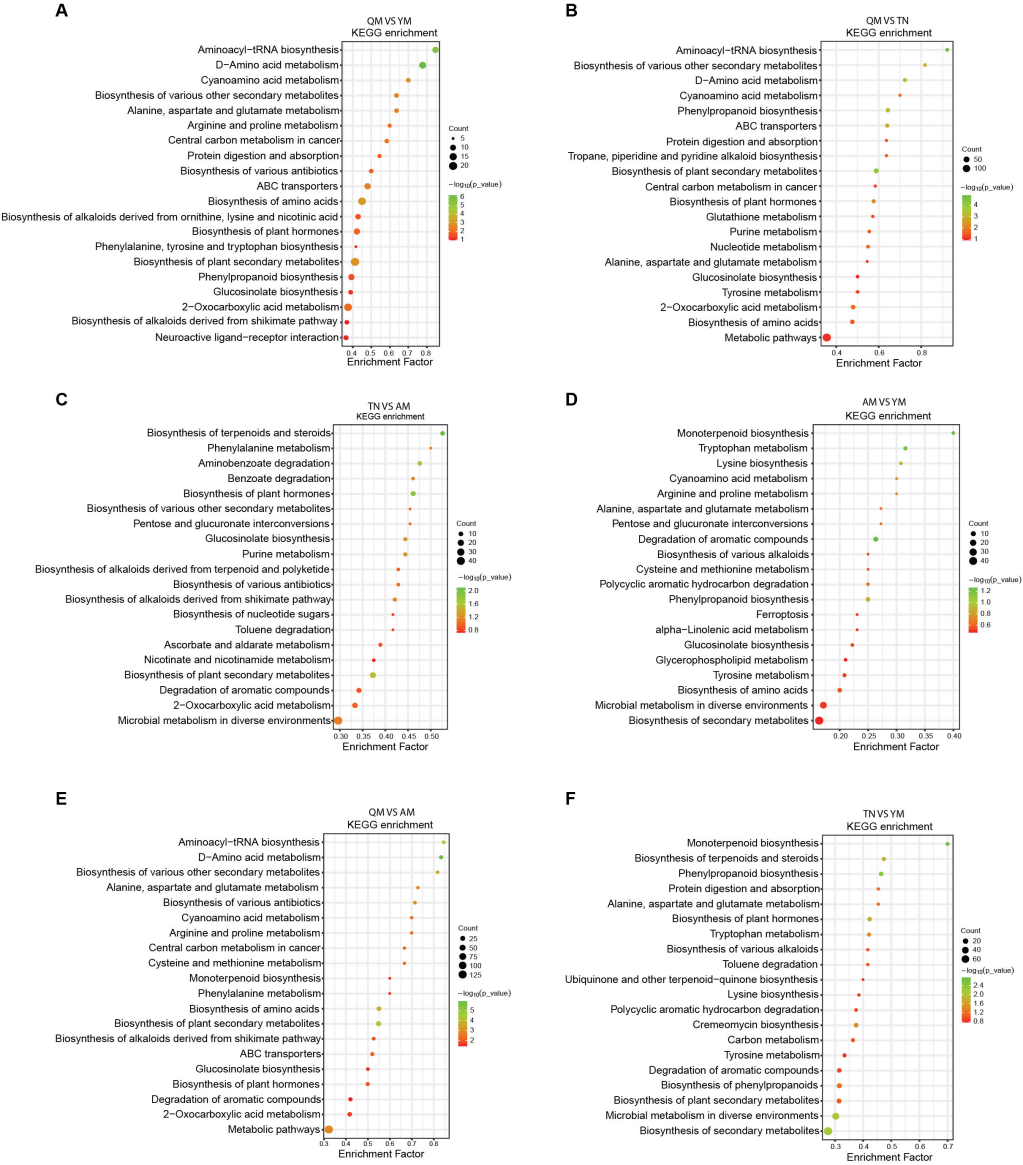
Dot plot visualization of KEGG pathway across four mango cultivars. Dot plots showing the metabolic pathway enrichment analysis results for *de novo* QM *vs*. YM **(A)**, QM *vs*. TN **(B)**, TN *vs* AM **(C)**, YM *vs*. AM **(D)**, QM *vs*. AM **(E)**, and YM *vs*. TN **(F)** using the KEGG database. The horizontal axis shows the negative decadic logarithm of the p-value, and the vertical axis shows the KEGG pathways sorted by decreasing significance from top. The color gradient from red to green reflects an increasing p-value and the size of each dot reflects the effect size for each pathway. QM, Qingmang; YM, Yumang; TN, Tainong; AM, Aomang.

Based on the HMDB database, the mango fruit-associated metabolites detected from the four distinct cultivars were annotated as carbohydrates and carbohydrate conjugates (12.37%), amino acids, peptides, and analogues (11.29%), fatty acids and conjugates (4.57%), flavonoid glycosides (4.3%), terpene glycosides (3.36%), sesquiterpenoids (3.09%), benzoic acids, and derivatives (2.96%), as the most abundant subclasses in the four mango cultivars (**Supplementary Figure S2**). Furthermore, the differential fruit metabolites from different mango cultivars based on KEGG mainly belonged to the class biosynthesis of secondary metabolites, microbial metabolism in diverse environments, biosynthesis of amino acids, degradation of aromatic compounds, biosynthesis of plant hormones, glucosinolate biosynthesis, ABC transporter, tyrosine metabolism, purine metabolism, glutathione metabolism, phenylpropanoid biosynthesis, phenylalanine, tyrosine and tryptophan biosynthesis, 2-oxocarboxylic acid metabolism, cremeomycin biosynthesis, and carbon metabolism (**Figures 4A–F**).

Specifically, when Yumang was compared to Qingmang, the top fold-change-enriched metabolites were 6-(methylsulfonyl)hexyl glucosinolate, mhppa sulfate, methyl 2,3,6-tri-O-galloyl-B-D-glucopyranoside, 1-dodecanoyl-sn-glycero-3-phosphocholine. While Qingmang was enriched with 5-(3’,4’,5’-trihydroxyphenyl)-gamma-valerolactone 4’-sulfate, tetramethylquercetin 3-rutinoside, N-gamma-glutamyl-S-trans-(1-propenyl)cysteine, kickxioside and pomiferin compared to Yumang (**Supplementary Figure S3A**). Similarly, the metabolites 6-(methylsulfonyl)hexyl glucosinolate, luteolin 7-O-[beta-D-glucuronosyl-(1->2)-beta-D-glucuronide], methyl 2,3,6-tri-O-galloyl-B-D-glucopyranoside and 4-methoxyglucobrassicin were enriched in Qingman compared with Tainong. While, the Tainong cultivar was enriched in the metabolites L-arginine, H-gamma-glutamyl-glutamine, ornithine and obacunone (**Supplementary Figure S3B**). Furthermore, the pair-wise comparison of top fold change metabolites revealed the enrichment of L-arginine, H-gamma-glutamyl-glutamine, ornithine, and 6’-O-galloyl salidroside in Qingman than in Aomang. In contrast, Aomang was enriched in icariside H1, 1-O-beta-D-glucopyranosyl-4-epiamplexine, xi-linalool 3-[rhamnosyl-(1-&Gt;6)-glucoside], and peonidin-3-O-arabinoside (**Supplementary Figure S3C**).

A comparison of Yumang and Tainong revealed enrichment of mhppa sulfate, quercetin-3-O-arabinoglucoside, sorbitan palmitate, lipoyllysine and dendronobiloside B. In contrast, Tainong was enriched in the metabolites tricetin, casoxin D, tetramethylquercetin 3-rutinoside, 4-methoxyglucobrassicin and 4’-methylepicatechin 5-glucuronide as compared to Yumang (**Supplementary Figure S3D**). The top fold-change-enriched metabolites in Aomang than Yumang were tricetin, casoxin D, phenylacetylaspartic acid and 4’-methylepicatechin 5-glucuronide, while in Yumang, they were mhppa sulfate, methyl 2,3,6-tri-O-galloyl-B-D-glucopyranoside, and (4-methylcyclohex-3-ene-1,1-diyl)dimethanol (**Supplementary Figure S3E**). Likewise, Tainong as compared to Aomang was also enriched in metabolites, such as methyl 2,3,6-tri-O-galloyl-B-D-glucopyranoside, 4-O-digalloyl-3,5-di-O-galloylquinic acid, 4-methoxyglucobrassicin and 4-methoxyglucobrassicin. Aomag was enriched in quercetin-3-O-arabinoglucoside, convallatoxin, crosatoside B and phenylacetylaspartic acid compared with that in Tainong (**Supplementary Figure S3F**).

### 3.3 Bacterial community diversity in the peel-pulp of different mango cultivars

**Figure 5:**
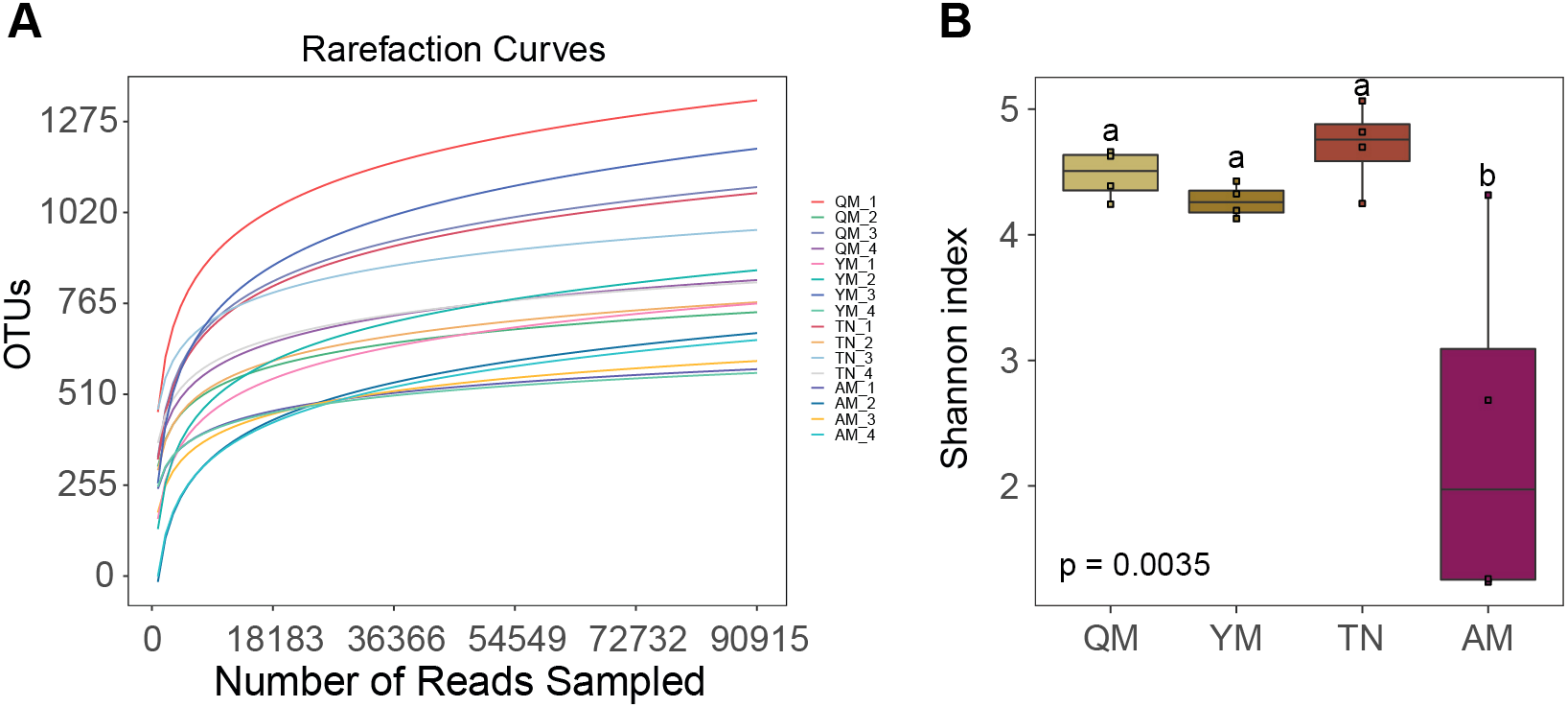
Bacterial community diversity in mango fruit collected from different cultivars. **(A)** Rarefaction curves of mango fruit samples obtained from the four mango cultivars. The x-axis shows the number of reads in each sample rarefied to 90,915 sequences per sample, and the y-axis indicates the number of OTUs detected based on the rarefied sequences in each sample. **(B)** Alpha diversity of fruit-associated bacterial communities in four distinct mango cultivars based on the Shannon diversity index. Different letters above each box indicate statistically significant differences according to the LSD test (p < 0.05). QM, Qingmang; YM, Yumang; TN, Tainong; AM, Aomang.

The four mango cultivars showed a distinct number of bacterial OTUs based on rarefied sequence counts (**Figure 5A**). The boxplot based on the mean values of the Shannon index showed that the alpha diversity of Tainong was higher than that of Qingmang and Yumang. The Aomang cultivar showed significantly lower Shannon index values than those of the other three cultivars (**Figure 5B**). Similarly, the Simpson index values followed the same pattern as the Shannon diversity for all four cultivars (**Supplementary Figure S4**).

**Figure 6:**
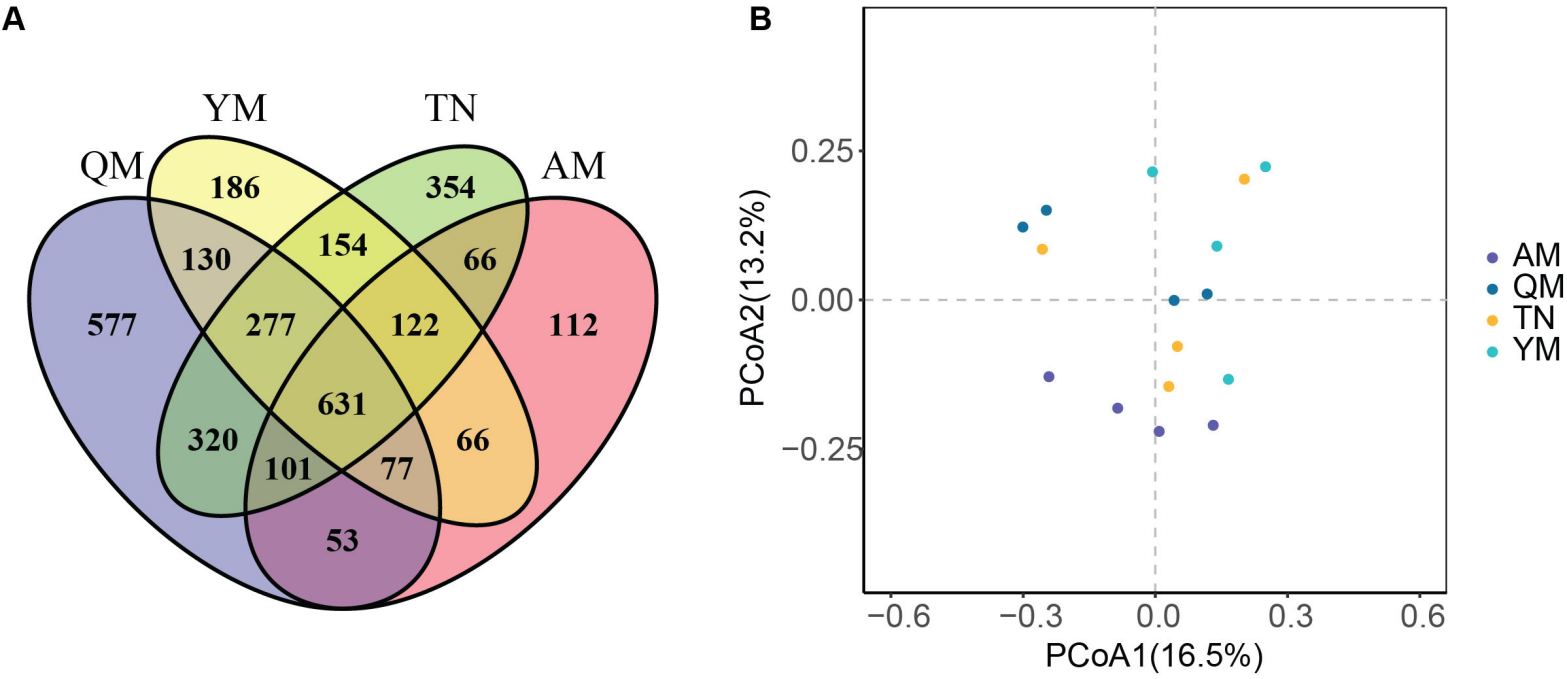
Variation in bacterial community structure across mango cultivars. **(A)** Venn diagram showing the unique and shared OTUs among the four mango cultivars. PCoA of the bacterial community structure showing differences in the distribution of samples along axis-1 and axis-2 based on unweighted-UniFrac distance. QM, Qingmang; YM, Yumang; TN, Tainong; AM, Aomang.

Analysis of the total bacterial OTUs across the four cultivars revealed significant differences in the number of OTUs. The cultivars Qingmang, Tainong, Yumang, and Aomang were inhabited by 1535, 1394, 1012 and 597 unique OTUs, respectively. A total of 631 OTUs were identified as core and shared OTUs across all cultivars (**Figure 6A**). Next, we conducted principal coordinate analysis (PCoA) of the unweighted UniFrac distance, a phylogenetic metric sensitive to changes in lineage presence and absence, to examine changes in the structure of fruit bacterial microbiota between different mango cultivars. We observed that the bacterial microbiota inhabiting the peel-pulp of Tainong, Aomang, Qingmang, and Yumang were moderately but significantly different from each other (PERMANOVA, p = 0.02). The PCoA1 first coordinate explained a total variation of 16.5% of the bacterial β-diversity among all cultivars. The second coordinate of PCoA 2 showed a variation of 13.2% in the bacterial β-diversity. Notably, the bacterial communities of Aomang and Yumang were clearly separated along PCoA Axis 2 (**Figure 6B**). These results imply that the mango genetics and biochemical properties of fruits create a niche that selectively enriches distinct sets of microbiota.

### 3.4 Differences in the composition of bacterial microbiota across mango cultivars

**Figure 7:**
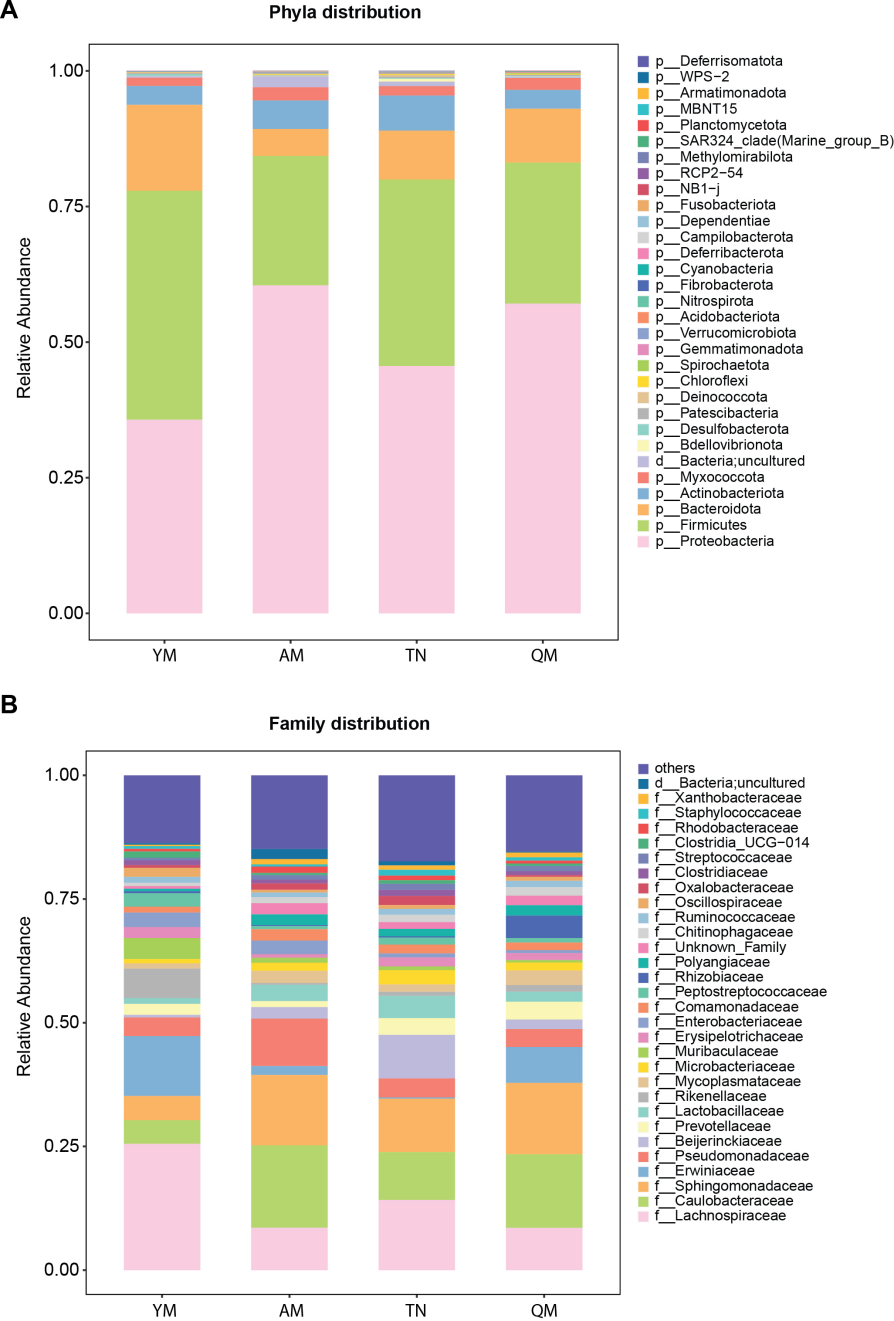
The composition of bacterial communities in mango fruits. Relative abundance of fruit-associated bacterial communities at phylum **(A)** and family **(B)** levels across four distinct cultivars. QM, Qingmang; YM, Yumang; TN, Tainong; AM, Aomang.

We identified a diverse number of bacteria belonging to different phyla in the fruits of four genetically distinct mango cultivars. Phyla Proteobacteria, Firmicutes, Bacteroidetes, Actinobacteria, and Myxococcota were dominant across the four cultivars. Specifically, the relative abundance of Proteobacteria was higher in Aomang and Qingmang than in Yumang and Tainong (**Figure 7A**). The abundance of Firmicutes was higher in Yumang and Tainong than that in Aomang and Qingmang. Bacteroidetes were more abundant in Yumang than in other cultivars. The relative abundance of Actinobacteria was significantly higher in Tainong than that in Qingmang and Yumang. The abundance of Acidobacteria was significantly higher in Qingmang and Tainong than in Yumang (**Figure 7A**).

At the family level, all cultivars showed a distinct enrichment of several bacterial taxa. The bacterial families Alcanivoracaceae and Catenulisporaceae were significantly enriched in Qingmang (**Figure 7B**). Similarly, the relative abundances of Ardenticatenaceae, Corynebacteriaceae, Geodermatophilaceae, Haliangiaceae, Weeksellaceae, and Xanthomonadaceae were higher in Aomang than in the other cultivars. The Yumang cultivar was enriched with the bacterial families Chromobacteriaceae, Marinifilaceae, Sphingomonadaceae, Sutterellaceae, and Xanthobacteraceae. The bacterial family Diplorickettsiaceae was more abundant in Tainong than in Aomang, Qingmang, or Yumang (**Figure 7B**).

Covering from the phylum to genus level, all cultivars showed distinct patterns of bacterial enrichment. The cultivar Aomang had the highest number of enriched bacterial genera, followed by Yumang, Tainong, and Qingmang, which is consistent with the results of cultivar separation in the PCoA. The bacterial genera enriched in Aomang were *Actinocatenispora*, *Actinomyces*, *Amaricoccus*, *Burkholderia*, *Caenimonas*, *Candidatus Arthromitus*, *Chryseobacterium*, *Cloacibacterium*, *Clostridioides*, *Corynebacterium*, *Enhydrobacter*, *Geodermatophilus*, *Haemophilus*, *Haliangium*, *Janthinobacterium*, *Paludicola*, *Pseudoxanthomonas*, *Rothia*, *Rubellimicrobium*, *Silanimonas*, and *Vulcaniibacterium* (**Supplementary Figure S5**). The fruits of the cultivar Yumang were enriched in the bacterial genera *Alistipes*, *Aquabacterium*, *Aurantisolimonas*, *Bilophila*, *Colidextribacter*, *Duganella*, *Enterorhabdus*, *Flectobacillus*, *Harryflintia*, *Lachnoclostridium*, *Lachnospiraceae*_NK4A136_group, *Lachnospiraceae*_UCG-010, *Larkinella*, *Odoribacter*, *Oscillibacter*, *Parasutterella*, *Patulibacter*, *Rikenella*, and *Vogesella* (**Supplementary Figure S5**). For the cultivar Tainong, the fruit-enriched bacterial genera were *Acidiphilium*, *Aquicella*, *Curtobacterium*, *Diaphorobacter*, *Quadrisphaera*, *Rhodoplanes*, and *Rosenbergiella* (**Supplementary Figure S5**). The relative abundance of bacterial genera *Alcanivorax*, *Catenulispora*, *Jiella*, *Komagataeibacter*, and *Thiopseudomonas* were greater in Qingmang than Aomang, Yumang and Tainong (**Supplementary Figure S5**). The abundances of the top 50 bacterial genera detected in the fruits of the four mango cultivars are shown in **Supplementary Figure S6**.

### 3.5 Correlation between key bacterial taxa and metabolites

**Figure 8:**
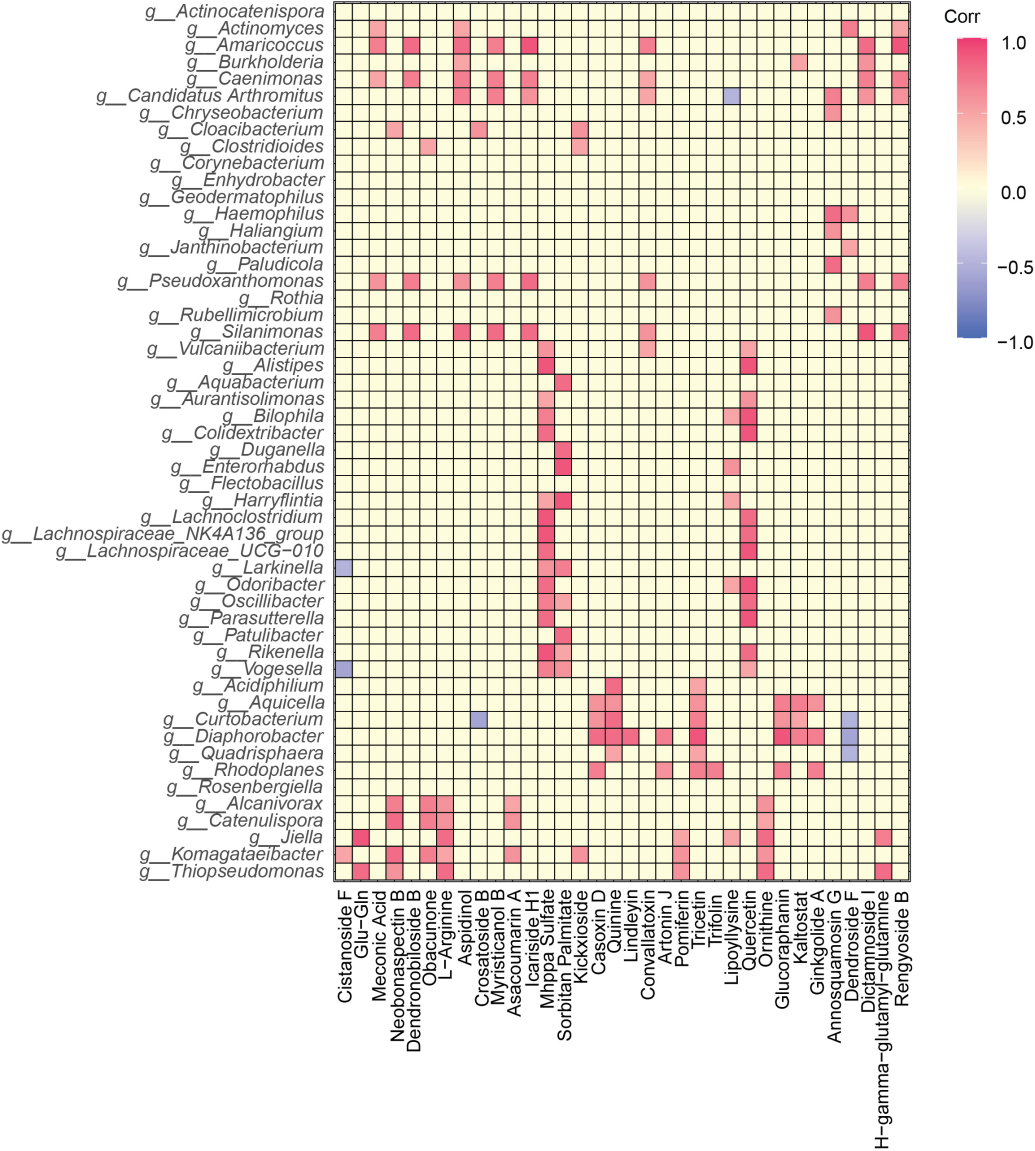
Correlation heatmap of fruit-associated differentially abundant bacterial genera and top-fold change metabolites based on Spearman’s correlation index values across four distinct mango cultivars. Correlation were considered significant when the *p*-value < 0.05 (*p < 0.05).

Several differentially abundant bacterial genera across mango cultivars were positively correlated with top fold-change metabolites. In the Qingmang cultivar, the bacterial genera *Alcanivorax*, *Catenulispora*, *Jiella*, *Komagataeibacter*, and *Thiopseudomonas* were positively correlated with the metabolites ornithine and L-arginine (**Figure 8**). Several metabolites, including neobonaspectin B, obacunone, pomiferin and asacoumarin A positively correlated with the bacterial genus *Komagataeibacter*. For the Tainong cultivar, *Acidiphilium*, *Aquicella*, *Curtobacterium*, *Diaphorobacter* and *Quadrisphaera* were positively correlated with tricetin and quinine (**Figure 8**). Casoxin D, glucoraphanin and kaltostat levels were positively correlated with the abundances of *Aquicella*, *Curtobacterium* and *Diaphorobacter*. The two metabolites mhppa sulfate and sorbitan palmitate were mainly correlated with most of the bacterial genera (*Alistipes*, *Aurantisolimonas*, *Bilophila*, *Colidextribacter*, *Lachnoclostridium*, *Lachnospiraceae*_NK4A136_group, *Lachnospiraceae*_UCG-010, *Odoribacter*, *Oscillibacter*, *Parasutterella*, *Rikenella*, and *Vogesella*) enriched in the Yumang cultivar (**Figure 8**). The metabolites in the fruits of Aomang (meconic acid, dendronobiloside B, aspidinol, myristicanol B, icariside H1, convallatoxin, dictamnoside I and rengyoside B) were positively correlated with *Silanimonas*, *Pseudoxanthomonas*, *Caenimonas* and *Amaricoccus*.

## 4 Discussion

Plant genetic diversity markedly shapes the physiological characteristics of mangoes owing to variations in genes that regulate distinct metabolic pathways. Mango cultivars, such as green, yellow, and red, are generally categorized based on their shape and peel color such as green, yellow, and red (Chen et al., 2022). This variation in mango peel color from green to red is associated with changes in the composition of two important pigment markers, anthocyanins and carotenoids (Ranganath et al., 2018). For instance, the carotenoid content was significantly increased in yellow-colored mangoes during fruit ripening (Ranganath et al., 2018), while the increased anthocyanin content was found to be associated with red-colored “Guifei” mangoes (Chen et al., 2022). Importantly, the market value and quality of mango fruit are highly dependent on these fruit color traits (Patel et al., 2024). In this study, we selected four mango cultivars with distinct peel colors (from green to red) to examine their metabolic profiles and microbiome composition. Initial investigations of mango peel-pulp revealed that these cultivars were markedly different in terms of their nutrient content. The cultivars Yumang and Qingmang had higher dry matter, crude protein, neutral detergent fiber, acid detergent fiber, lignin, and water-soluble carbohydrate contents than the other two cultivars. It has been postulated that the dry weight of fruits mainly consists of cell walls and carbohydrates (Roch et al., 2020), and can vary significantly among different cultivars (Raigón et al., 2008; Arivalagan et al., 2012). Similarly, fruits contain proteins, lignin, and carbohydrates, and their levels can vary between different cultivars/species or fruit development stages (Colombié et al., 2015; Zhang et al., 2020).

Mango fruits are a potent source of specialized metabolites (Maldonado-Celis et al., 2019). Mango pulp contains amino acids, anthocyanins, pectin, polyphenols, sugars, and vitamins, and mango peel is composed of functional compounds such as β-carotene, mangiferin, and protocatechuic acids, which are recognized as antimicrobial, anti-diabetic, and anti-inflammatory (Lebaka et al., 2021). Our analysis of peel-pulp metabolomics revealed cultivar-specific metabolite compositions in the mango cultivars Tainong, Aomang, Qingmang, and Yumang. This aligns with previous observations that plant phenotypes and genetics can significantly influence metabolite composition, thereby influencing fruit chemistry and nutritional quality (Fiehn, 2002; Tharanathan et al., 2006; Li et al., 2020, 2022).

At the metabolite level, we identified abundant subclasses, such as carbohydrates and carbohydrate conjugates(12.37%), amino acids, peptides, and analogues (11.29%), fatty acids and conjugates (4.57%), flavonoid glycosides (4.3%), terpene glycosides (3.36%), sesquiterpenoids (3.09%), and benzoic acids and derivatives (2.96%) in four mango cultivars. Mangoes are a rich source of carbohydrates owing to their fundamental role in fruit metabolism (Dar et al., 2016; Reddy and Reddy, 2005). The presence of glucose conjugates, including derivatives such as caffeoyl-, coumaroyl-, and galloyl-glucose, as well as nucleotide-sugar intermediates such as UDP-glucose and CDP-glucose, in mango peel-pulp underscores the significant role of carbohydrate metabolism beyond energy storage. It is integral to complex biosynthetic pathways associated with the production of phenolic and secondary metabolites (Farag et al., 2022). Furthermore, the differential abundance of amino acid derivatives, such as L-arginine and ornithine, among mango cultivars reflects variations in nitrogen metabolism, potentially influencing fruit development and stress response capacities (Shu et al., 2020; Pandey et al., 2015). It has been hypothesized that during the late developmental stages of peach, the lignification process is deemed complete, and therefore a high abundance of amino acids is utilized either as an energy source or as a precursor for the synthesis of flavonoids (Lombardo et al., 2011). Interestingly, we also observed key compounds belonging to flavonoid biosynthesis in mango, such as tricetin, quercetin-3-O-arabinoglucoside, tetramethylquercetin 3-rutinoside, 4’-methylepicatechin 5-glucuronide, luteolin 7-O-[beta-D-glucuronosyl-(1->2)-beta-D-glucuronide] and peonidin-3-O-arabinoside, which may have implications for both fruit color, antioxidant capacity, and pathogen resistance (Yang et al., 2025; Sivankalyani et al., 2016). These differences in metabolite composition of carbohydrates and carbohydrate conjugates, amino acids, and flavonoids may explain differences in fruit quality attributes such as flavor, aroma, and nutritional value among the four mango cultivars.

Fruit-associated microorganisms are important components of the phyllosphere microbiome and play critical roles in carbohydrate metabolism, fruit storability, and disease suppression (Saminathan et al., 2018; Kusstatscher et al., 2020; Wu et al., 2019). We observed the dominance of Phylum Proteobacteria, Actinobacteria, Bacteroidetes, Acidobacteria and Firmicutes in different mango cultivars. The prevalence and varying levels of Proteobacteria in mango fruits across different cultivars may be attributed to the availability of different quantities of carbon sources, such as amino acids, carbohydrates, and lipids. These carbon sources facilitate bacterial growth and allow them to adjust to the evolving environmental conditions within the fruit as it matures (Peighamy-Ashnaei et al., 2006; Kazakov et al., 2009; Xia et al., 2015). Similarly, earlier studies have observed a high abundance of Firmicutes, Actinobacteria, Acidobacteria, and Bacteroidetes in the flesh of melon fruit and grapes (Glassner et al., 2015; ZarraonaIndia et al., 2015). Importantly, the analysis of bacterial genera in the peel-pulp of mango fruits showed the enrichment of several genera involved in the biocontrol of a diverse range of plant pathogens. For example, *Burkholderia pyrrocinia* produces volatile organic compounds (VOCs) that enhance the ability of fruits to resist and inhibit fungal pathogens (Wang et al., 2025). Similarly, the genus *Janthinobacterium* produces the antifungal pigment violacein, which inhibits fungal pathogens (Xia et al., 2021). The genus *Komagataeibacter* produces bacterial cellulose, which forms biofilms on fruit surfaces. These biofilms facilitate microbial colonization, reduce the pathogen load, and influence the fruit ripening process (Augimeri et al., 2015). Surprisingly, we also detected the enrichment of *Clostridioides* in mango peel-pulp, which is a significant human pathogen that causes severe intestinal infections (Bernabe et al., 2024). The presence of *Clostridioides* in the peel-pulp of mango fruit is likely due to environmental contamination caused by the widespread prevalence of bacteria in soil, combined with the anaerobic microenvironments within the fruit tissue that favor its survival and germination. This underscores the potential of *Clostridioides* spores to survive in fruits during harvest, handling, and storage. Although the presence of *Clostridioides* in mangoes does not necessarily indicate a direct health risk, improved hygiene and handling practices are recommended to reduce microbial contamination and to ensure fruit safety.

The interplay between plant-associated microbiota and metabolites is critical for fruit quality and health. Microbial communities influence plant metabolism by affecting the synthesis of primary and secondary metabolites (Hussain et al., 2025). The bacterial microbiota can trigger the production of beneficial metabolites in plants through the synthesis of bioactive compounds (Sun et al., 2024). Interestingly, plant microbiota can also suppress certain metabolic pathways involved in plant defense and stress tolerance (Colaianni et al., 2021). In this study, the correlations observed between specific microbial genera and metabolites in mango fruits were stronger, highlighting that fruit-associated microbiota may influence host physiological processes by regulating metabolic diversity, thereby affecting mango health. For example, the correlation of amino acids L-arginine and ornithine, as well as secondary metabolites, such as flavonoids and alkaloids, with specific bacteria may contribute to plant defense and stress resistance (Winter et al., 2015; Jiang et al., 2024; Bag et al., 2022; Chen et al., 2021). This suggests that the interplay between microbiota and metabolites may enhance the ability of mangoes to resist environmental stressors. This also underscores the potential of microbiome management as a strategy to enhance plant resilience in mango (Hussain et al., 2026). A thorough understanding of microbial-metabolite interactions under various environmental conditions in these mango cultivars may offer new opportunities to improve the quality of mango fruit production.

## 5 Conclusion

This study demonstrated that mango cultivars with different genetic backgrounds selectively shaped fruit-associated metabolic profiles and bacterial community composition. Detailed analysis of fruit metabolites from the four mango cultivars revealed a diverse set of differentially abundant metabolites associated with functions such as carbohydrates, amino acids, flavonoid biosynthesis, phenolics, and secondary metabolites. Integrated analysis identified cultivar-specific signatures between the bacterial communities and differentially accumulated key metabolites. The positive correlation between bacterial genera and metabolites, such as L-Arginine, Ornithine, and tricetin, has strong implications for fruit development and (a)biotic stress resistance. These results provide a scientific basis for future targeted management of the fruit microbiome to modulate the metabolic composition of mangoes to enhance desirable traits, such as fruit color, flavor, and shelf life.

## Data Availability

The datasets presented in this study can be found in online repositories. The names of the repository/repositories and accession number(s) can be found below: https://www.ncbi.nlm.nih.gov/, PRJNA1367298.
