# Supplementary material for: Deciphering the interplay between fruit-associated metabolites and bacterial communities across four distinct mango cultivars
Source: Front Plant Sci. 2026 Feb 6;17:1754579. doi: 10.3389/fpls.2026.1754579 (PMC12920474; doi:10.3389/fpls.2026.1754579)
Supplement: Supplementary file 1 [file DataSheet1.doc]

**Supplementary materials**

**Deciphering the interplay between fruit-associated metabolites and bacterial communities across four distinct mango cultivars**

Chuanfang Zhang^1,2,3^, Rong Wan^1,2,*^, Siwei Nong^1,2^, Wei Huang^1,2^, Fengzhen Wang^1,2^, Zhengzhou Yang^1,2^, Zhengjie Zhu^1,2^

^1^Guangxi Key Laboratory of Biology for Mango, College of Agriculture and Food Engineering, Baise University, Baise, China

^2^College of Subtropical Characteristics, Agricultural Industry, Baise, China

^3^Sichuan Vocational and Technical College, Suining, Sichuan, China

**^*^Corresponding author:** Rong Wan (wanrong@bsuc.cn)­

**Supplementary figures: 6**

**
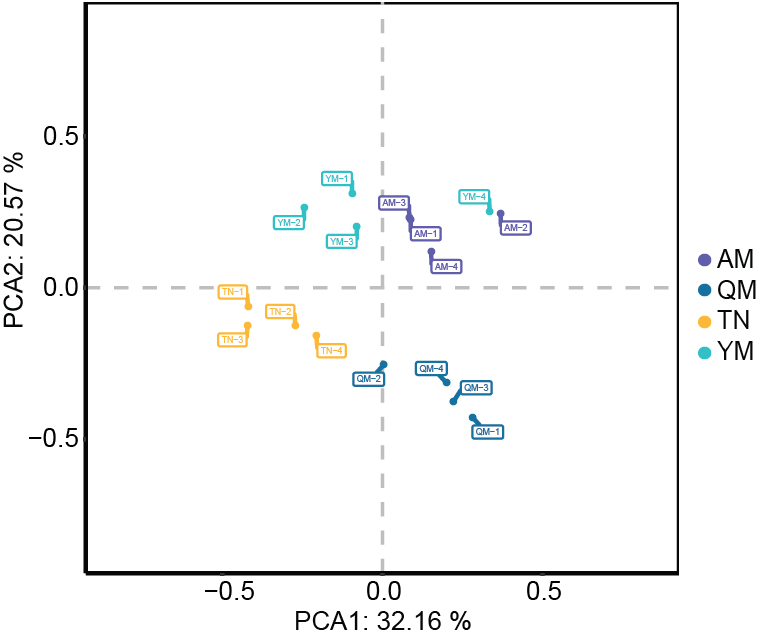
**

**Figure S1.** Principle component analysis (PCA) of fruit-associated metabolites from four distinct mango cultivars. QM, Qingmang; YM, Yumang; TN, Tainong; and AM, Aomang.

**
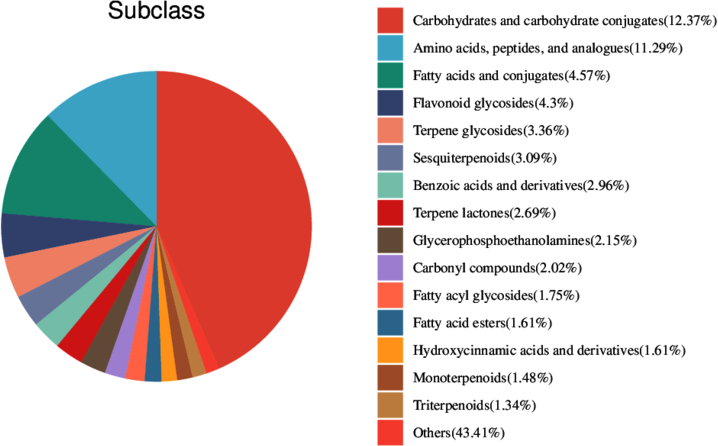
**

**Figure S2.** Pie-chart of metabolites classes annotated to the detected fruit-associated metabolites from four distinct cultivars in the HMDB database.

**
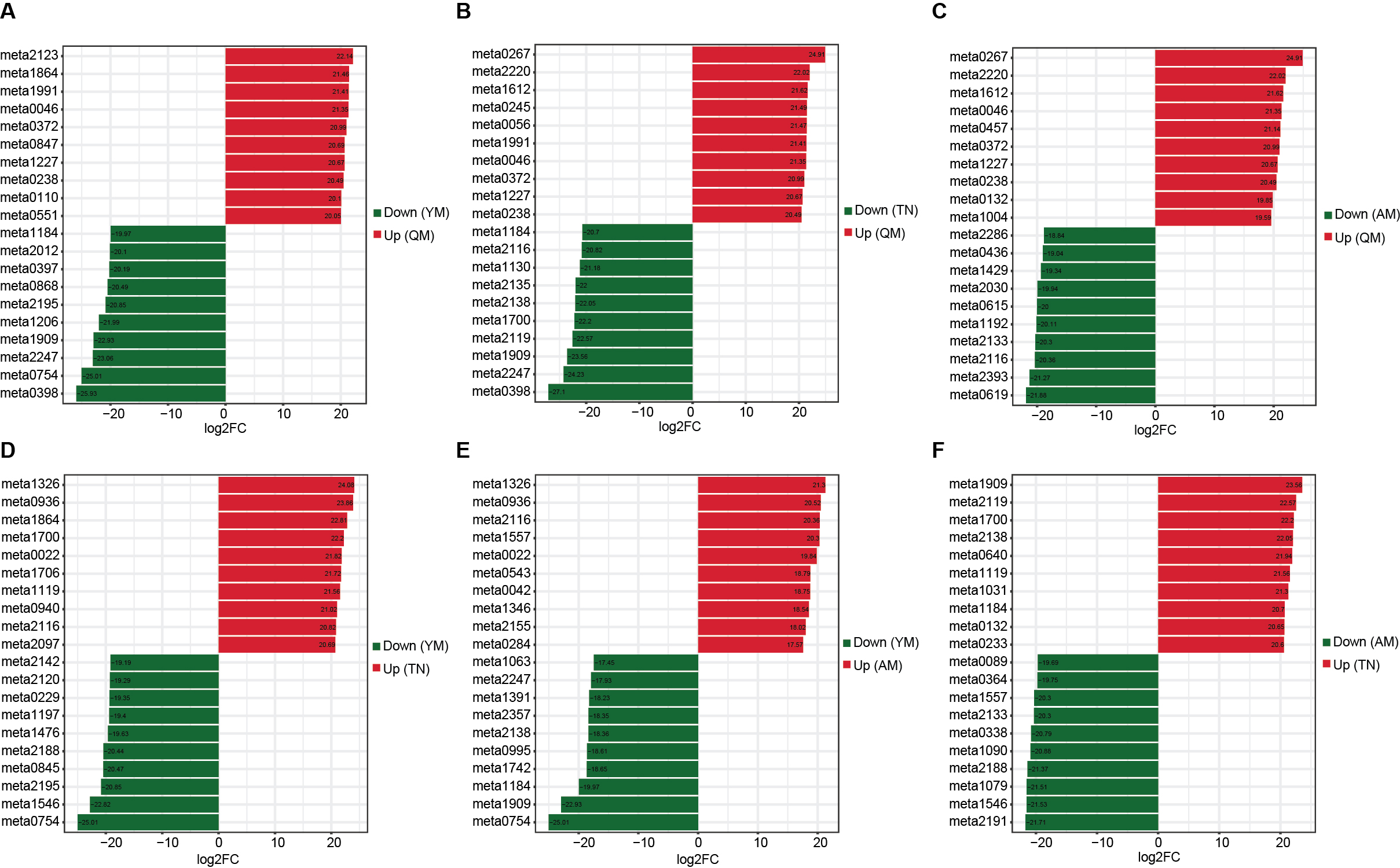
**

**Figure S3.** Pair-wise comparison of top fold change fruit metabolites between distinct cultivars. QM, Qingmang; YM, Yumang; TN, Tainong; and AM, Aomang.


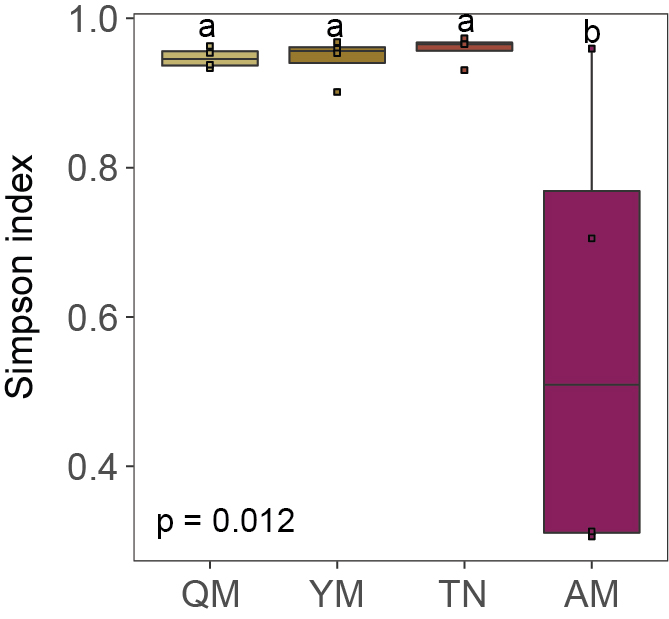


**Figure S4.** Alpha-diversity of fruit-associated bacterial communities in four distinct mango cultivars based on Simpson diversity index. Different letters above each box indicate statistically significant differences according to the LSD test (p < 0.05). QM, Qingmang; YM, Yumang; TN, Tainong; and AM, Aomang.

**
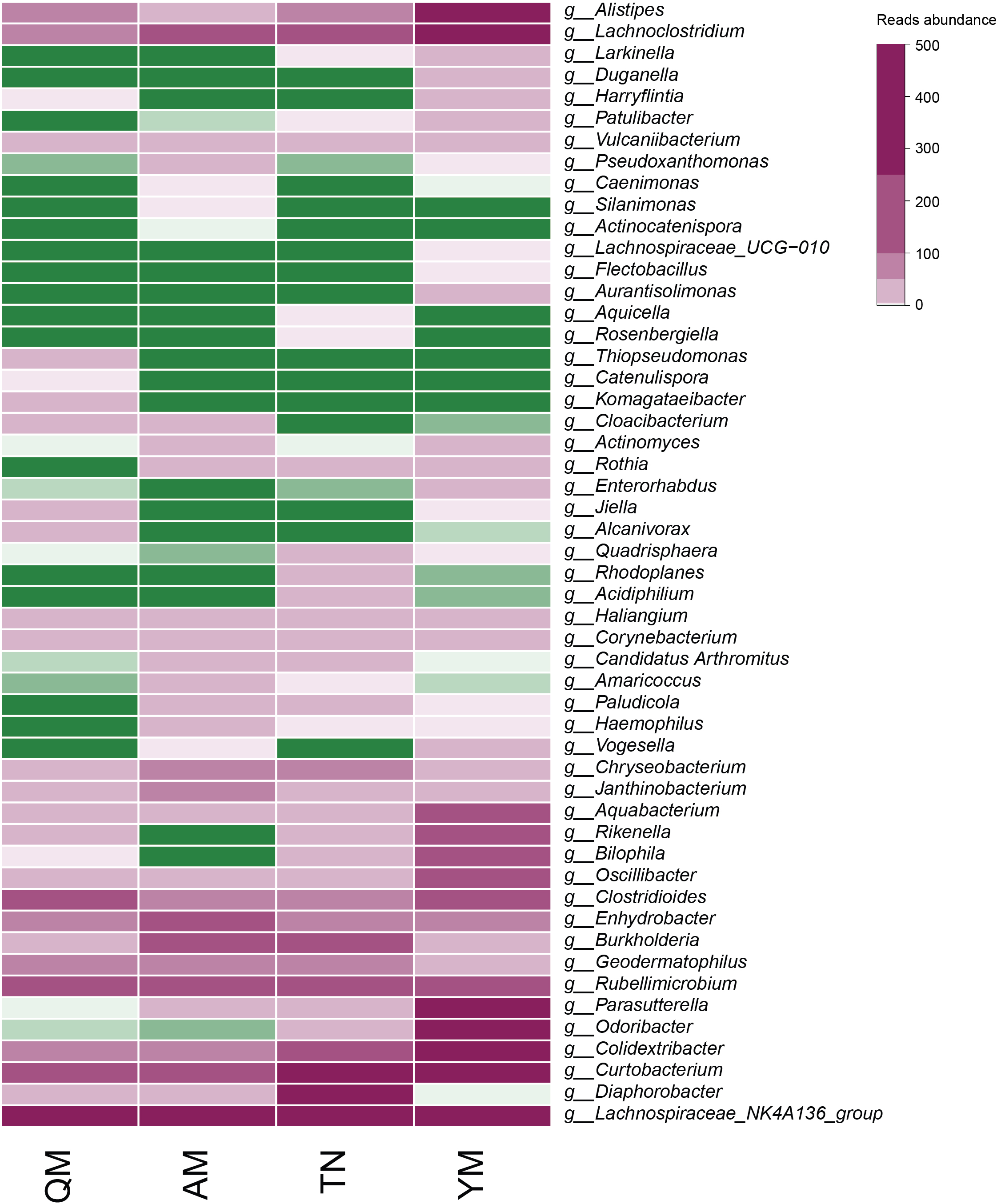
**

**Figure S5.** Differentially abundant bacterial genera in fruits of four distinct mango cultivars. Statistically significant differences was observed according to the LSD test (p < 0.05). QM, Qingmang; YM, Yumang; TN, Tainong; and AM, Aomang.

**
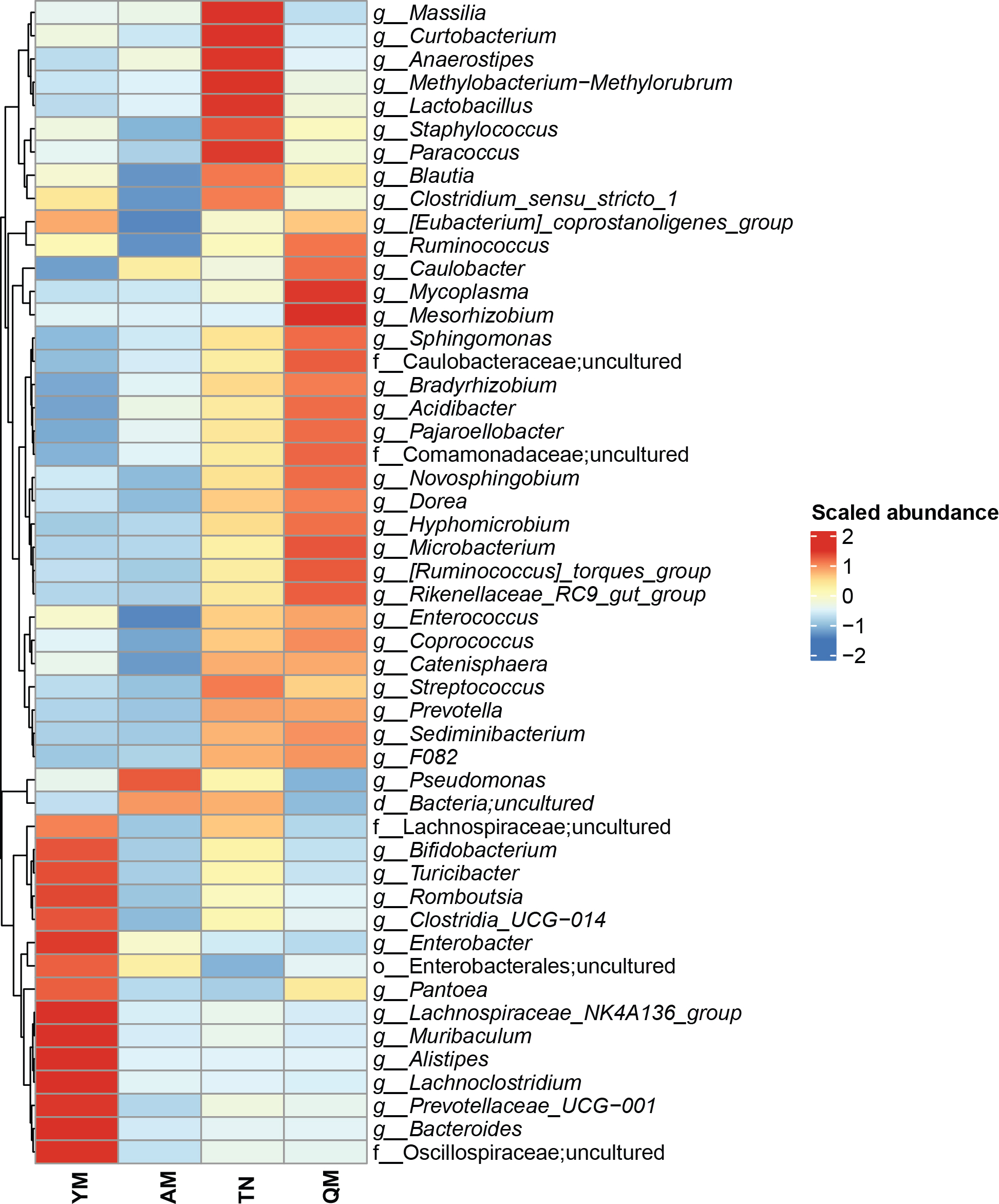
**

**Figure S6.** The relative abundance of top 50 bacterial genera observed in fruits across four mango cultivars. QM, Qingmang; YM, Yumang; TN, Tainong; and AM, Aomang.
